# Development of a nutritional management protocol for patients with severe traumatic brain injury: a Delphi study

**DOI:** 10.3389/fnut.2026.1904179

**Published:** 2026-09-17

**Authors:** Zhixue Ye, Jinxia Rong, Kejing Yuan, Neng Zhou, Lina Feng, Qinhong Xu

**Affiliations:** 1 Emergency Intensive Care Unit, Department of Emergency Medicine, The First Affiliated Hospital of Ningbo University, Ningbo, Zhejiang, China; 2 Emergency Ward, Department of Emergency Medicine, The First Affiliated Hospital of Ningbo University, Ningbo, Zhejiang, China; 3 Department of Nursing, The First Affiliated Hospital of Ningbo University, Ningbo, Zhejiang, China

**Keywords:** Delphi, evidence-based, nutrition, protocol, severe traumatic brain injury

## Abstract

**Background:**

Patients with severe traumatic brain injury (sTBI) commonly experience hypermetabolism, catabolism, and feeding intolerance, making nutritional management an essential part of clinical care. However, there is still a lack of standardized and integrated protocols to guide nutritional management in this population.

**Aims:**

To develop an evidence-based nutrition management protocol for patients with sTBI.

**Methods:**

An evidence-based approach was adopted to formulate the protocol. Relevant evidence was identified through systematic retrieval, followed by quality appraisal, evidence synthesis, and grading. A preliminary protocol was then developed and further revised through two rounds of Delphi expert consultation. Experts rated each item in terms of feasibility and importance.

**Results:**

A total of 17 studies were included after evidence retrieval and appraisal and the preliminary protocol consisting of 5 primary indicators, 10 secondary indicators, and 33 tertiary indicators was developed. This preliminary protocol was then refined through two rounds of Delphi expert consultation involving 15 experts. The Kendall’s harmony coefficients for item feasibility were 0.399 and 0.331, respectively, and those for item importance were 0.288 and 0.465, respectively. The final protocol included 4 primary indicators, 11 secondary indicators and 33 tertiary indicators.

**Conclusion:**

This study developed an evidence-based nutritional management protocol for patients with sTBI. The protocol provides an evidence-informed framework to support standardized nutritional management in patients with sTBI.

## 1 Introduction

Traumatic brain injury (TBI) is a major global health problem and remains one of the leading causes of death and long-term disability worldwide (1, 2). It may result from a range of causes, most commonly road traffic incidents, falls, violence, and sports- or work-related injuries (3). Nearly 50% of the global population will sustain at least one traumatic brain injury during their lifetime (4). TBI encompasses a spectrum of injury severity, ranging from mild to severe forms. Although more than 90% of TBI cases presenting to hospital are classified as mild (defined as a Glasgow Coma sum score of 13–15) (3), severe traumatic brain injury (sTBI) contributes disproportionately to mortality, intensive care utilization, and persistent functional impairment (5, 6). As advances in neurocritical care have improved early survival after the initial insult, optimizing supportive strategies to mitigate secondary injury has become an important clinical priority.

## 2 Background

sTBI is frequently accompanied by marked hypermetabolism and hypercatabolism (7), driven by neuroendocrine stress, systemic inflammation, and altered substrate utilization. These metabolic disturbances accelerate protein breakdown, contribute to negative nitrogen balance, and are often accompanied by hyperglycemia and markedly increased energy demands (8). In the acute phase after TBI, energy requirements may rise to approximately 100–200% of baseline-predicted resting energy expenditure (REE), and this hypermetabolic state may persist for weeks to months depending on injury severity and the degree of recovery (9, 10). In addition, factors such as gastrointestinal dysfunction, hemodynamic instability, temperature regulation, sedation, mechanical ventilation, and feeding intolerance often complicate the delivery of nutritional support in this population (10, 11). As a result, patients with sTBI admitted to the intensive care unit (ICU) are highly vulnerable to inadequate energy and protein delivery during the acute phase of critical illness, leading to substantial nutritional deficits even when nutritional support is provided (12). Such deficits may occur despite nutritional support and can contribute to cumulative nutritional insufficiency during ICU stay. Adequate nutritional support is crucial in patients with TBI, as it contributes to the maintenance of metabolic and cellular homeostasis, helps preserve gastrointestinal homeostasis via the brain–gut axis, and may reduce mortality and the incidence of infectious complications (13–15).

Although nutritional support is a core part of neurocritical care, providing adequate and individualized nutrition for sTBI patients remains inconsistent in clinical practice. Evidence suggests that personalized nutritional formulas can offer clinical advantages over standard options for patients with severe head injuries (16). However, controversy remains regarding several core components of nutritional management. Consensus is still lacking on several key clinical issues, including the optimal timing of enteral versus parenteral nutrition, the appropriate level of protein supplementation, and the definitive clinical benefits of immunomodulating components (17). These uncertainties contribute to variation in management strategies across different clinical settings. Moreover, current recommendations are often dispersed across multiple guidelines and reviews. There is still a lack of a coherent and standardized bedside protocol for nutritional management in patients with sTBI.

Therefore, there is a need to develop a structured and clinically applicable nutritional management protocol for patients with sTBI based on the best available evidence. This study aimed to develop an evidence-based nutritional management protocol for patients with sTBI using an evidence-based approach, and to further assess the feasibility and importance of the protocol through Delphi expert consultation. The protocol will provide a reference for nutritional management in clinical patients with sTBI.

## 3 Methods

### 3.1 Study design

The study was conducted in two phases: Development of a nutrition management protocol for patients with sTBI based on a comprehensive evidence synthesis; Finalization of the protocol through two rounds of Delphi expert consultation. The study was conducted and reported in accordance with the Guidance on Conducting and REporting DElphi Studies (CREDES).

### 3.2 Literature research and selection criteria

Following the “5S” evidence pyramid model (18), a top-down search strategy was adopted. Clinical decision support resources included BMJ Best Practice and UpToDate. Guideline repositories and professional society resources included the National Institute for Health and Care Excellence (NICE) guideline repository, Guidelines International Network (GIN), American Society for Parenteral and Enteral Nutrition (ASPEN), European Society for Clinical Nutrition and Metabolism (ESPEN), Society of Critical Care Medicine (SCCM), European Society of Intensive Care Medicine (ESICM), American College of Surgeons (ACS), Chinese Medical Association, and yimaitong. For these resources, a targeted topic-based search was conducted using the terms “traumatic brain injury/head trauma/nutrition”. English and Chinese bibliographic databases searched included PubMed, Cochrane Library, the Joanna Briggs Institute (JBI) Evidence-Based Healthcare Database, Web of Science, CINAHL, SinoMed, China National Knowledge Infrastructure (CNKI), Wanfang, and VIP Database. The search period covered from database inception to November 2025. The full search strategies for all English and Chinese bibliographic databases are provided in Supplementary Table S1.

The inclusion criteria for the literature were: (1) Guidelines, clinical practice, evidence summaries and systematic reviews. (2) Providing guiding opinions on nutrition support for patients with sTBI. (3) Chinese or English language.

The exclusion criteria for the literature were: (1) Duplicate publications or articles with unavailable full text; (2) Abstracts, comments, letters, or non-evidence-based reviews; (3) Animal studies.

All literature searches were conducted manually on the native platforms of the respective databases and evidence resources using predefined search strategies. No automated search or filtering software was used. Retrieved records were imported into EndNote 21 for reference management and duplicate removal, and subsequent study screening was performed manually by the researchers.

### 3.3 Quality appraisal and evidence synthesis

The methodological quality of the included literature was appraised according to study type. Guidelines were evaluated using the Appraisal of Guidelines for Research and Evaluation II (AGREE II) (19), while systematic reviews and meta-analyses were appraised using the JBI Critical Appraisal Checklist for Systematic Reviews and Research Syntheses (20). Clinical decision support resources from authoritative databases were regarded as high-quality evidence. For evidence summaries, the original references were traced and appraised using the appropriate JBI critical appraisal tools based on study design.

Two researchers independently completed the quality appraisal and evidence extraction. Disagreements were resolved through discussion, with consultation from a third researcher when required. During synthesis, higher-quality and more recent evidence was prioritized; complementary evidence was integrated, and evidence with concordant recommendations was combined. Recommendations specifically applicable to sTBI were preferentially extracted. When sTBI-specific evidence was insufficient, recommendations from general adult critical care evidence that were considered more applicable to the clinical characteristics of patients with sTBI were used as supplementary evidence. Based on this synthesis, evidence statements with direct practical relevance were translated into preliminary protocol items for subsequent Delphi consultation. Evidence levels were assigned according to the JBI Levels of Evidence and Grades of Recommendation (2014 version) (21), ranging from Level 1a to Level 5c.

### 3.4 Expert panel

To ensure the clinical feasibility and scientific rigor of the sTBI nutritional management protocol, experts from tertiary-level (Grade A) hospitals in Shanghai and Zhejiang Province, China, were invited to participate in the Delphi survey. The inclusion criteria for the participating experts were as follows: (1) currently practicing in fields related to neurocritical care, clinical medicine, specialized nutrition, or nursing management; (2) Possessing at least 10 years of clinical experience in relevant fields; (3) holding a bachelor’s degree or higher; (4) Volunteered for the study.

### 3.5 Delphi survey

Two rounds of the Delphi survey were conducted between December 2025 and March 2026. A flow chart illustrating the Delphi process is provided in Supplementary Figure S2. The questionnaires were developed in Microsoft Word and distributed to experts via email or WeChat. Experts were requested to complete and return the questionnaires within 2 weeks. The questionnaire consisted of three sections. (1) A brief overview of the research background, objectives, and detailed instructions for completing the survey. (2) A structured evidence table where experts assessed each item using a 5-point Likert scale. Scores ranged from 1 (“very unimportant/infeasible”) to 5 (“very important/feasible”), with additional space provided for qualitative comments or revision suggestions. These qualitative comments were reviewed and summarized by the research team after each round and were used to guide item modification, merging, deletion, or splitting, as appropriate. (3) Expert base information: The general information of the experts includes gender, age, working years, education level, professional title, work city, work unit, work field, etc. Additionally, experts were asked to rate their familiarity with the topic on a scale of 0.2 (very unfamiliar) to 1 (very familiar), and to indicate the basis of their judgment. There are four types of judgment basis: practical experience, theoretical analysis, reference to domestic and foreign literature, and intuition selection, along with the degree of high, medium, or low influence.

### 3.6 Statistical analysis

Statistical analyses were conducted using SPSS 26.0 software (IBM Corp; Armonk, United States). Mean and standard deviation (SD) were used to summarize the feasibility and importance ratings because mean scores and coefficients of variation (CVs) were predefined as part of the Delphi item-retention criteria, and previous methodological evidence has shown that the use of mean and SD for Likert-scale data remains robust even when the data deviate from normality or are treated as ordinal rather than interval-level measurements (22). The criteria for retaining items were defined as a mean ≥ 4.0 and a CV ≤ 0.25. The expert positivity was evaluated by the response rate of questionnaires. A response rate above 70% was regarded as acceptable. The expert authority coefficient (CR) was calculated as the mean of the experts’ judgment basis (Ca) and their familiarity with the research protocol (Cs), using the formula: CR = (Ca + Cs)/2. The level of agreement among experts was assessed using Kendall’s coefficient of concordance (Kendall’s W). Kendall’s W values were interpreted according to the following criteria: <0.30, weak agreement; 0.30–0.49, moderate agreement; 0.50–0.69, good agreement; and ≥0.70, strong agreement (23).

### 3.7 Ethics consideration

This study was approved by the Institutional Review Boards of the First Affiliated Hospital of Ningbo University. Written informed consent was obtained from all experts before participation in the study.

## 4 Results

### 4.1 Literature search results

A total of 826 records were identified through the systematic search. After removing duplicates (*n* = 162), screening titles and abstracts (*n* = 621), 783 articles were excluded. Following full-text review, 26 articles were excluded due to irrelevant content, ineligible study type, or animal studies, and 17 articles were finally included. The included articles consisted of 6 guidelines, 3 clinical decisions, 7 meta-analyses and 1 evidence summary. The detailed study selection process is presented in Supplementary Figure S1. The basic characteristics of the included literature are presented in Supplementary Table S2. Among the included guidelines, those appraised were rated as grade A or B and were all retained for evidence synthesis. The included systematic reviews were also considered methodologically acceptable. Detailed appraisal results are presented in Supplementary Tables S3, S4.

### 4.2 Basic information of experts

A total of 15 experts participated in our study, including 7 experts in nursing, 3 in critical care medicine, 2 in nutrition, 2 in neurosurgery, and 1 in emergency medicine. Table 1 shows the basic characteristics of experts.

**Table 1:** Basic characteristics of experts.

Items	Number	Percent
Gender		
Male	7	46.67%
Female	8	53.33%
Age		
30 ~ 39	2	13.33%
40 ~ 49	11	73.33%
≥50	2	13.33%
Working years		
11 ~ 15	4	26.67%
≥16	11	73.33%
Professional title		
Deputy senior	8	53.33%
Senior	7	46.67%
Education level		
Bachelor’s degree	5	33.33%
Master’s degree	8	53.33%
Doctoral degree	2	13.33%
Fields		
Nursing	7	46.67%
Critical Care Medicine	3	20.00%
Nutrition	2	13.33%
Neurosurgery	2	13.33%
Emergency Medicine	1	6.67%

### 4.3 Delphi consultation results

#### 4.3.1 Enthusiasm and authority of the experts

A two-round expert consultation was conducted. In the first round, 16 questionnaires were distributed and 15 were returned. The questionnaire response rates were 93.75% in the first round and 100% in the second round. The expert authority coefficient (Cr) was calculated using the judgment basis coefficient (Ca) and the familiarity coefficient (Cs), according to the formula Cr = (Ca + Cs)/2. The Cr in the two Delphi rounds were 0.90 and 0.91, respectively, indicating a high level of authority among the participating experts.

#### 4.3.2 Degree of coordination of the expert opinions

The CV for the feasibility ratings in the first round ranged from 0 to 0.32, while that for importance ranged from 0 to 0.24. In the second round, the mean scores for all items were ≥ 4.0, with the CV for feasibility ranged from 0 to 0.17 and that for importance ranged from 0 to 0.13. The coefficients of variation decreased in the second round, indicating improved consistency in expert opinions. In addition, the Kendall’s W values ranged from 0.292 to 0.456 across the two rounds, and all values were statistically significant (*p* < 0.001), indicating a satisfactory level of consensus among the experts and good reliability of the evaluation results (Table 2).

**Table 2:** Coordination of expert consultations in the two rounds.

Round	Feasibility			Importance		
	Kendall’s W	χ2	*P*-value	Kendall’s W	χ2	*P*-value
First Round	0.399	281.378	*P* < 0.001	0.288	202.692	*P* < 0.001
Second Round	0.331	233.569	*P* < 0.001	0.465	327.748	*P* < 0.001

### 4.4 Delphi round

In the first round of expert consultation, 9 experts provided a total of 30 suggestions for revision. Based on these comments, the research team revised the draft protocol through four approaches: modification, merging, deletion and item splitting. When differing views arose regarding the incorporation of expert suggestions, decisions were made through team discussion with reference to the supporting evidence. Specifically, 8 items were modified, 1 item was deleted, 2 items were merged, and 3 items were split. The primary indicators “Nutritional screening and assessment” and “timing of nutrition initiation” were merged into one domain: “Nutritional screening, pre-initiation assessment, and decision-making.” The original secondary item, “Immunomodulatory and Enteral Nutrition Formula,” was split into two items: “Supplemental Parenteral Nutrition Strategy” and “Selection of Immunomodulatory and Enteral Formulas”. The former focuses on decisions regarding the route and timing of nutritional support, whereas the latter addresses formula composition and indications for use, thereby improving the structural clarity and logical coherence of the framework. The detailed information on tertiary indicators revisions is provided in Supplementary Table S2.

In the second round, six experts provided six suggestions. One expert suggested that the method for assessing gastric residual volume should be further clarified. After discussion and review of the relevant literature, the research team supplemented the original item by adding the operational description that gastric residual volume may be assessed by aspiration through the gastric or enteral tube using a 50- or 60-mL syringe, thereby improving the standardization and clinical applicability of the item. In addition, based on the experts’ suggestions, we further refined the item on post-pyloric feeding. For patients with contraindications to nasal tube insertion, such as skull base fracture or nasal cavity injury, the oral route was added as an alternative option to enhance the safety and applicability of the item in special populations. Finally, based on the experts’ suggestions, the wording of four items was further optimized.

After two rounds of expert consultation, consensus was reached among the experts, and the final protocol was composed of 4 primary indicators, 11 secondary indicators and 33 tertiary indicators. A concise version of the final protocol is presented in Table 3, and the complete protocol, including the 11 secondary indicators, detailed recommendations, and expert consensus ratings for feasibility and importance, is provided in Supplementary Table S6.

**Table 3:** Nutritional support management protocol for patients with severe traumatic brain injury.

No.	Recommendation topic	Recommendation
I. Organizational support and team management		
1	Multidisciplinary team	Establish a multidisciplinary nutritional support team for patients with sTBI.
2	Standardized procedures and quality control	Use standardized procedures, defined role responsibilities, regular training, and quality control.
II. Screening, assessment, and initiation decisions		
3	Nutritional risk screening	Perform nutritional risk screening within 24 h of admission and repeat screening after 1 week or when major clinical changes occur.
4	Screening tools	Use NRS-2002 or NUTRIC score for nutritional risk screening.
5	Glycemic and metabolic assessment	Assess blood glucose at admission and dynamically evaluate glycemic metabolism during artificial nutrition.
6	Comprehensive nutrition assessment	Conduct multidimensional nutritional assessment; serum biomarkers should not be used as the sole indicator.
7	Contraindication screening	Screen for contraindications before initiating EN or PN.
8	Early EN initiation	Early EN within 48 h is recommended for patients without contraindications.
9	EN after shock	Low-dose EN may be initiated after hemodynamic stabilization in patients with shock.
10	EN during therapeutic hypothermia	During therapeutic hypothermia, low-dose EN may be advanced according to tolerance and rewarming.
11	PN initiation decision	PN timing should be individualized when EN is contraindicated or insufficient.
12	Early PN when required	Early PN may be considered when parenteral support is clinically required.
III. Nutritional intervention strategy		
13	Feeding route and tube placement	Post-pyloric feeding is preferred; alternative access may be considered when nasal tube insertion is contraindicated.
14	Delivery method	Continuous EN should be delivered using a controllable feeding pump.
15	Energy requirement estimation	IC is preferred for assessing energy requirements. If unavailable, estimate EE using VCO₂/VO₂-based methods or 20–25 kcal/kg/d, with individualized adjustment.
16	Stepwise target achievement	Nutritional delivery should be advanced stepwise toward target requirements.
17	High-risk or malnourished patients	Earlier achievement of energy and protein targets may be considered in high-risk or severely malnourished patients.
18	Protein target	Protein targets should be individualized according to renal function, metabolic status, and tolerance.
19	Supplemental PN	SPN should be individualized according to nutritional risk, disease severity, and EN tolerance.
20	SPN timing threshold	SPN may be considered when EN remains insufficient to meet energy and protein targets, with individualized adjustment.
21	Immunomodulating formula	Immunomodulating formulas may be combined with standard EN according to clinical status.
22	Selected immune nutrients and probiotics	Selected immune nutrients or probiotics may be considered for appropriate patients.
23	Glutamine supplementation	Glutamine may be considered in selected patients with preserved hepatic and renal function.
24	Fiber supplementation	Routine fiber supplementation is not recommended in patients with severe gastrointestinal dysmotility or feeding intolerance.
IV. Implementation, monitoring, and complication management		
25	Nurse-led implementation	A nurse-led EN protocol should be implemented with ongoing monitoring of tolerance and complications.
26	Position management	The head of the bed should be elevated in intubated patients receiving EN unless contraindicated.
27	Tolerance monitoring	Feeding tolerance should be assessed regularly using a standardized approach.
28	GRV monitoring	GRV monitoring should be symptom-guided rather than routine in patients without gastrointestinal distress.
29	Blood glucose monitoring	Blood glucose should be monitored during early artificial nutrition and adjusted according to clinical stability.
30	Insulin titration monitoring	During initial insulin titration, monitor blood glucose more frequently and reduce frequency after target achievement and dose stabilization.
31	Glucose target	Intensive glucose control is not recommended; insulin should be used when glucose exceeds the target range.
32	Refeeding syndrome prevention	Electrolytes should be monitored during early EN to prevent and manage refeeding syndrome.
33	Feeding intolerance management	When marked feeding intolerance occurs, EN should be slowed or suspended, causes evaluated, and prokinetic therapy considered when appropriate.

NRS-2002, Nutritional Risk Screening 2002; NUTRIC, Nutrition Risk in the Critically Ill; EN, enteral nutrition; PN, parenteral nutrition; IC, indirect calorimetry; REE, resting energy expenditure; SPN, supplemental parenteral nutrition; GRV, gastric residual volume.

## 5 Discussion

This study developed an evidence-based nutritional management protocol for patients with sTBI based on the best available evidence and refined it through two rounds of Delphi expert consultation. The final protocol comprised 4 primary indicators, 11 secondary indicators, and 33 tertiary indicators, covering the key domains of nutritional management in sTBI: organizational support and team management; nutritional screening; pre-initiation assessment, and decision-making, nutritional interventions; implementation monitoring with complication management.

During evidence integration, priority was given to high-level and recently published evidence, which helped ensure the scientific rigor and timeliness of the preliminary protocol. The robustness of this protocol was further supported by the multidisciplinary composition of the expert panel. In this study, the experts were drawn from multiple disciplines, which helped ensure the comprehensiveness and relevance of the protocol. All experts had more than 10 years of clinical experience in their respective fields, providing a solid professional basis for evaluating the scientific validity and practical applicability of the protocol. After two rounds of Delphi consultation, the CV for feasibility range from 0 to 0.32 in the first round and from 0 to 0.17 in the second round, while the CV for importance ranged from 0 to 0.24 and from 0 to 0.13, respectively. Kendall’s W values for feasibility were 0.399 and 0.331 in the two rounds, respectively, and those for importance were 0.288 and 0.465, with all *p* values <0.001, indicating a satisfactory degree of consensus among the experts.

It is also noteworthy that several recommendations received relatively lower feasibility or importance scores, particularly those related to PN initiation, rapid achievement of nutritional targets, immunomodulating components, glutamine supplementation, and intensive electrolyte monitoring. This may reflect the fact that these items are more dependent on individual clinical conditions, local resources, and ongoing uncertainty in the evidence base. In addition, the feasibility ratings of some recommendations may have been influenced by the realities of busy clinical settings, where implementation often requires more intensive monitoring, closer multidisciplinary coordination, or greater staffing and resource support. Nevertheless, because they still met the predefined retention criteria and were considered, after expert feedback and final team discussion, to represent important observation and decision-making points in the nutritional support of patients with sTBI, they were retained in order to preserve the clinical completeness of the protocol and to reflect areas that require individualized judgment in practice.

Nutritional intervention in sTBI remains challenging because the challenge is not simply to provide nutrients, but to deliver the right amount, through an appropriate route, at an appropriate time, and with a formula that can be tolerated under unstable physiological conditions. EN is generally preferred because it supports gut integrity and is more physiological, and many guidelines recommend early EN when clinically feasible (10, 24, 25). However, in neurocritical care, the adequacy of EN is further compromised by delayed gastric emptying, feeding intolerance, and the technical difficulty of post-pyloric tube placement. At the same time, the marked hypermetabolic and hypercatabolic response after injury makes underfeeding particularly harmful (26). More than half of patients with sTBI may develop malnutrition or be at high risk of malnutrition during ICU stay (27), and many fail to achieve even basic energy or protein intake within the first few days of critical care (28).

In this context, the role of PN as an adjunct to early EN, as well as the optimal timing of its initiation, remains controversial in patients with sTBI (29). Although accumulating evidence suggests that combined EN and PN may have positive effects on clinical outcomes in patients with TBI when enteral feeding alone is insufficient (30, 31), this does not support a uniform strategy of early parenteral supplementation for all patients. Recommendations on when to initiate exclusive PN or add supplemental PN remain conditional and highly dependent on nutritional risk, EN feasibility, and the adequacy of EN delivery. For patients in whom EN is not feasible, several guidelines and evidence sources (level of evidence: 1a) support initiating exclusive PN as early as possible in those at high nutritional risk or with severe malnutrition (24, 32, 33). Meanwhile, supplemental PN is generally considered after 7–10 days when EN provided less than 60% of energy and protein requirements (24). These considerations underscore the importance of individualized nutritional strategies in sTBI. Beyond total caloric provision, the choice of formula, the use of high-protein support, and the potential role of specific functional components such as omega-3 fatty acids, glutamine, or probiotics may all need to be considered in relation to metabolic demand, gastrointestinal tolerance, and the patient’s evolving clinical condition. For SPN, formulation should be adjusted according to EN intake, individualized energy and protein targets, renal and hepatic function, glycemic control, triglyceride level, lipid clearance, and overall metabolic tolerance. Given the hypercatabolic and hypermetabolic characteristics of sTBI, protein/nitrogen and amino acid provision through SPN should be considered in relation to the gap between actual EN intake and individualized protein targets, and adjusted according to renal and hepatic function, nitrogen balance when available, and metabolic tolerance (34, 35). In patients with high nutritional risk or persistently inadequate EN delivery, SPN may be used to improve protein and energy provision; in patients with renal or hepatic dysfunction, impaired glucose control, hypertriglyceridemia, or poor lipid clearance, the proportions of amino acids, glucose, and lipid emulsions should be adjusted more cautiously with laboratory monitoring (36). Therefore, SPN formulation should be individualized, and these component-level considerations were not incorporated as fixed recommendation statements in the final protocol.

Failure to achieve nutritional targets in sTBI is often related not only to the initial prescription itself, but also to interruptions in EN, poor feeding tolerance, inconsistent monitoring, and delayed responses to complications after feeding has been initiated. In this context, a nurse-led nutritional management protocol may offer advantages (37). Because bedside nurses are continuously involved in feeding delivery, symptom observation, tolerance assessment, blood glucose and gastric residual volume monitoring, and multidisciplinary communication, they are well positioned to identify deviations early and respond promptly to emerging problems. Glucose management in patients with sTBI should also consider high-risk subgroups, particularly patients with preexisting diabetes or preadmission hyperglycemia. Some observational studies have suggested that glycemic targets adjusted according to premorbid glycemic status, such as hemoglobin A1c (HbA1c), may be associated with outcomes in critically ill patients (38). However, the level of evidence remains limited. Current glycemic management guidelines for critically ill patients indicate that (39), for patients with preexisting diabetes or preadmission hyperglycemia, evidence from randomized controlled trials remains insufficient to make a formal recommendation regarding individualized glycemic targets. Therefore, rather than defining separate fixed glucose targets for these subgroups, our protocol emphasizes closer blood glucose monitoring and individualized management during nutritional support, especially in patients with diabetes, steroid therapy, osmotherapy, significant glucose fluctuations, or unstable neurological/metabolic status. Such an approach may help reduce unnecessary interruptions in EN, promote achievement of nutritional targets, and ultimately contribute to improved clinical outcomes (37, 40).

### 5.1 Limitation

This study has several limitations. First, because part of the evidence base was derived from adult critical care nutrition guidelines rather than guidance specifically developed for patients with sTBI, the applicability of some recommendations to the sTBI population may need to be further explored in future studies. Second, although the Delphi method is authoritative, it has potential for expert selection bias, and the regional characteristics of the expert panel, together with the limited number of nutrition specialists, may limit the applicability of the protocol in broader settings. Third, although the protocol was developed from the best available evidence and further refined through two rounds of Delphi expert consultation, with satisfactory expert ratings for feasibility and importance, it has not yet undergone prospective validation in real-world clinical practice.

### 5.2 Relevance to clinical practice

The clinical application value of this protocol lies in its ability to translate fragmented evidence and recommendations into a structured and practical framework for bedside nutritional management in patients with sTBI. By providing clear guidance on screening, initiation, route selection, target setting, monitoring, and complication management, the protocol may help reduce practice variation and support more standardized nutritional care. In addition, it may facilitate multidisciplinary collaboration, nurse-led implementation, and quality improvement in neurocritical care settings.

### 5.3 Recommendations for further research

Future research should prospectively assess the effectiveness of this protocol in clinical practice, with particular attention to objective outcomes such as nutritional attainment rates, adverse event rates, and long-term clinical outcomes. Further work is also needed to optimize and update the protocol as new evidence emerges, particularly for controversial aspects of nutritional management in sTBI.

## 6 Conclusion

This study established a nutritional management scheme for patients with sTBI, comprising 4 primary indicators, 11 secondary indicators, and 33 tertiary indicators, with expert consensus achieved. The developed scheme may provide a useful reference for clinical nutritional management in patients with sTBI. Future prospective studies are warranted to evaluate the clinical feasibility of this protocol and its impact on objective patient outcomes.

## Data Availability

The original contributions presented in the study are included in the article/Supplementary material, further inquiries can be directed to the corresponding author.
