# Supplementary material for: Development of a nutritional management protocol for patients with severe traumatic brain injury: a Delphi study
Source: Front Nutr. 2026 Sep 17;13:1904179. doi: 10.3389/fnut.2026.1904179 (PMC13626899; doi:10.3389/fnut.2026.1904179)
Supplement: Supplementary file 1 [file Data_Sheet_1.pdf]

Supplementary——CREDES checklist

Items of reporting	Reported on Section
Purpose and rationale. The purpose of the study should be clearly defined and demonstrate the appropriateness of the use of the Delphi technique as a method to achieve the research aim. A rationale for the choice of the Delphi technique as the most suitable method needs to be provided.	Introduction Section
Expert panel. Criteria for the selection of experts and transparent information on recruitment of the expert panel, sociodemographic details including information on expertise regarding the topic in question, (non)response and response rates over the ongoing iterations should be reported.	Methods Section 3.4
Procedure. Flow chart to illustrate the stages of the Delphi process, including a preparatory phase, the actual 'Delphi rounds', interim steps of data processing and analysis, and concluding steps.	Supplementary Figure S2
Definition and attainment of consensus. It needs to be comprehensible to the reader how consensus was achieved throughout the process, including strategies to deal with non-consensus.	Results Section 4.3
Results. Reporting of results for each round separately is highly advisable in order to make the evolving of consensus over the rounds transparent. This includes figures showing the average group response, changes between rounds, as well as any modifications of the survey instrument such as deletion, addition or modification of survey items based on previous rounds.	Results Section 4.3and supplementary
Discussion of limitations. Reporting should include a critical reflection of potential limitations and their impact of the resulting guidance.	Discussion Section 5.1
Adequacy of conclusions. The conclusions should adequately reflect the outcomes of the Delphi study with a view to the scope and applicability of the resulting practice guidance.	Conclusion Section
Publication and dissemination. The resulting guidance on good practice in palliative care should be clearly identifiable from the publication, including recommendations for transfer into practice and implementation. If the publication does not allow for a detailed	Not addressed as specific to palliative care research recommendations.

presentation of either the resulting practice guidance or the methodological features of the applied Delphi technique, or both, reference to a more detailed presentation elsewhere should be made (e.g. availability of the full guideline from the authors or online; publication of a separate paper reporting on methodological details and particularities of the process (e.g. persistent disagreement and controversy on certain issues)). A dissemination plan should include endorsement of the guidance by professional associations and health care authorities to facilitate implementation.	

1. Jünger S, Payne SA, Brine J, Radbruch L, Brearley SG. Guidance on Conducting and REporting DElphi Studies (CREDES) in palliative care: Recommendations based on a methodological systematic review. Palliat Med. 2017;31: 684–706.

doi:10.1177/0269216317690685
