# Supplementary material for: Development of a nutritional management protocol for patients with severe traumatic brain injury: a Delphi study
Source: Front Nutr. 2026 Sep 17;13:1904179. doi: 10.3389/fnut.2026.1904179 (PMC13626899; doi:10.3389/fnut.2026.1904179)
Supplement: Supplementary file 2 [file Table_1.docx]

***Supplementary Materials***

**Supplementary Table S1.** Search strategy

**Supplementary Table S2.** Basic characteristics of the included literature

**Supplementary Table S3.** Quality assessment results of the included guidelines

**Supplementary Table S4.** Quality assessment results of the included systematic reviews

**Supplementary Table S5.** Suggestions of the First Round of Expert Consultation

**Supplementary Table S6.** Detailed Nutritional Support Management Protocol for Patients with Severe Traumatic Brain Injury

**Supplementary Figure S1.** Flow diagram of the literature selection process

**Supplementary Figure S2.** Delphi survey processes

**Table S1.** Search strategy

| Database | Search strategy |
| --- | --- |
| PubMed | #1 Brain Injuries, Traumatic[MeSH Terms] OR brain injuries[MeSH Terms] OR Cerebral Hemorrhage, Traumatic[MeSH Terms] OR Craniocerebral Trauma[MeSH Terms] |
|  | #2 "TBI"[Title/Abstract] OR "Severe TBI"[Title/Abstract] OR "Craniocerebral Injury"[Title/Abstract] OR "head injury"[Title/Abstract] OR "head trauma"[Title/Abstract] OR "Severe Traumatic Brain Injury"[Title/Abstract] OR "Severe Head Injury "[Title/Abstract] OR "Severe Craniocerebral Trauma*"[Title/Abstract] OR "Brain Injurie*"[Title/Abstract] |
|  | #3 #1 OR #2 |
|  | #4 "Nutrition Support"[Mesh] OR "Nutrition Therapy"[Mesh] OR "Enteral Nutrition"[Mesh] OR "diet therapy"[Mesh] OR "Parenteral Nutrition"[Mesh] |
|  | #5 "enteral tube feeding"[Title/Abstract] OR "Enteral Nutrition"[Title/Abstract] OR "Nutritional Management"[Title/Abstract] OR "Nutrition Protocol"[Title/Abstract] OR "Nutrition Strategy"[Title/Abstract] OR "Parenteral Nutrition"[Title/Abstract] OR "nasogastric feeding"[Title/Abstract] OR "Nutrition Support*"[Title/Abstract] OR "Nutrition Therap*"[Title/Abstract] OR "Enteral Nutrition"[Title/Abstract] OR "diet therap*"[Title/Abstract] OR "Nutrition"[Title/Abstract] |
|  | #6 #4 OR #5 |
|  | #7 "review, systematic"[Mesh] OR "Meta analysis" [Mesh] |
|  | #8 "practice"[Title/Abstract] OR "recommendation"[Title/Abstract] OR "evidence summary"[Title/Abstract] OR "guideline"[Title/Abstract] OR "systematic review"[Title/Abstract] |
|  | #9 #7 OR #8 |
|  | #10 #3 AND #6 AND #9 |
| Web of Science | #1 TS=(TBI OR "Severe TBI" OR "Craniocerebral Injury" OR "head injury" OR "head trauma" OR "Severe Traumatic Brain Injury" OR "Severe Head Injury "OR "Severe Craniocerebral Trauma*" OR "Critical Head Trauma" OR "Brain Injurie*" OR "Cerebral Hemorrhage" or "brain injury" or "brain hemorrhage" or "traumatic brain injury") |
|  | #2 TS=("enteral tube feeding" OR "Enteral Nutrition" OR "Nutritional Management" OR "Nutrition Protocol" OR "Nutrition Strategy" OR "Parenteral Nutrition" OR "nasogastric feeding" OR "Nutrition Support*" OR "Nutrition Therap*" OR "Enteral Nutrition" OR "diet therap*" OR "Nutrition") |
|  | #3 TS=("practice" OR "recommendation" OR "evidence summary" OR guideline OR “systematic review" OR "Meta analysis") |
|  | #4 #1 AND #2 AND #3 |
| Cochrane Library | #1 MeSH descriptor: [brain injuries] explode all trees |
|  | #2 MeSH descriptor: [Intracranial Hemorrhages] explode all trees |
|  | #3 MeSH descriptor: [Craniocerebral Trauma] explode all trees |
|  | #4 MeSH descriptor: [Brain Injuries, Traumatic] explode all trees |
|  | #5 MeSH descriptor: [Cerebral Hemorrhage] explode all trees |
|  | #6 (TBI OR "Severe TBI" OR "Craniocerebral Injury" OR "head injury" OR "head trauma" OR "Severe Traumatic Brain Injury" OR "Severe Head Injury " OR "Severe Craniocerebral Trauma" OR "Critical Head Trauma" OR "Brain Injurie" OR "Cerebral Hemorrhage"):ti,ab,kw |
|  | #7 MeSH descriptor: [nutritional support] explode all trees |
|  | #8 MeSH descriptor: [diet therapy] explode all trees |
|  | #9 ("enteral tube feeding" OR "Enteral Nutrition" OR "Nutritional Management" OR "Nutrition Protocol" OR "Nutrition Strategy" OR "Parenteral Nutrition" OR "nasogastric feeding" OR "Nutrition Support" OR "Nutrition Therapy" OR "Enteral Nutrition" OR "diet therapy" OR "Nutrition"):ti,ab,kw |
|  | #10 #1 OR #2 OR #3 OR #4 OR #5 OR #6 |
|  | #11 #7 OR #8 OR #9 |
|  | #12 #10 AND #11 |
| CINAHL | #1 MH "Brain Injuries+" OR MH "Intracranial Hemorrhage+" OR MH "Cerebral Hemorrhage+" OR MH "Head Injuries+" |
|  | #2 TI (TBI OR "Severe TBI" OR "Craniocerebral Injury" OR "head injury" OR "head trauma" OR "Severe Traumatic Brain Injury" OR "Severe Head Injury "OR "Severe Craniocerebral Trauma*" OR "Critical Head Trauma" OR "Brain Injurie*" OR "Cerebral Hemorrhage") OR AB (TBI OR "Severe TBI" OR "Craniocerebral Injury" OR "head injury" OR "head trauma" OR "Severe Traumatic Brain Injury" OR "Severe Head Injury "OR "Severe Craniocerebral Trauma*" OR "Critical Head Trauma" OR "Brain Injurie*" OR "Cerebral Hemorrhage") |
|  | #3 MH "Nutritional Support+" OR MH "Nutritional Assessment" OR MH "Nutritional Requirements+" OR MH "Diet Therapy+" |
|  | #4 TI ("enteral tube feeding" OR "Enteral Nutrition" OR "Nutritional Management" OR "Nutrition Protocol" OR "Nutrition Strategy" OR "Parenteral Nutrition" OR "nasogastric feeding" OR "Nutrition Support" OR "Nutrition Therapy" OR "Enteral Nutrition" OR "diet therapy" OR "Nutrition") OR AB ("enteral tube feeding" OR "Enteral Nutrition" OR "Nutritional Management" OR "Nutrition Protocol" OR "Nutrition Strategy" OR "Parenteral Nutrition" OR "nasogastric feeding" OR "Nutrition Support" OR "Nutrition Therapy" OR "Enteral Nutrition" OR "diet therapy" OR "Nutrition") |
|  | #5 MM "Practice Guidelines" OR MM "Systematic Review" OR MM "Meta Analysis" |
|  | #6 TI (“practice” OR “recommendation” OR “evidence summary” OR guideline OR “systematic review” OR “Meta analysis”) OR AB (“practice” OR “recommendation” OR “evidence summary” OR guideline OR “systematic review” OR “Meta analysis”) |
|  | #7 #1 OR #2 |
|  | #8 #3 OR #4 |
|  | #9 #5 OR #6 |
|  | #10 #7 AND #8 AND #9 |
| CNKI | (SU=重症颅脑损伤 + 重症颅脑创伤 + 重症颅脑外伤 + 重型颅脑损伤 + 严重颅脑损伤 + 创伤性脑损伤 + 脑外伤 + 脑损伤 + 脑出血) AND (SU=营养 + 营养管理 + 营养治疗 + 营养支持 + 营养疗法 + 营养方案 + 肠内营养 + 肠道营养 + 鼻饲 + 管饲 + 管道喂养 + 鼻肠管喂养 + 胃管喂养) AND (SU=指南 + 系统综述 + 证据总结 + Meta分析 + 实践) |
| Wanfang | (（主题:(重症颅脑损伤 OR 重症颅脑创伤 OR 重症颅脑外伤 OR 重型颅脑损伤 OR 严重颅脑损伤 OR 创伤性脑损伤 OR 脑外伤 OR 脑损伤 OR 脑出血) or 题名或关键词:(重症颅脑损伤 OR 重症颅脑创伤 OR 重症颅脑外伤 OR 重型颅脑损伤 OR 严重颅脑损伤 OR 创伤性脑损伤 OR 脑外伤 OR 脑损伤 OR 脑出血)） and （主题:(营养 OR 营养管理 OR 营养治疗 OR 营养支持 OR 营养疗法 OR 营养方案 OR 肠内营养 OR 肠道营养 OR 鼻饲 OR 管饲 OR 管道喂养 OR 鼻肠管喂养 OR 胃管喂养) or 题名或关键词:(营养 OR 营养管理 OR 营养治疗 OR 营养支持 OR 营养疗法 OR 营养方案 OR 肠内营养 OR 肠道营养 OR 鼻饲 OR 管饲 OR 管道喂养 OR 鼻肠管喂养 OR 胃管喂养)） and 主题:(指南 OR 系统综述 OR 证据总结 OR Meta分析 OR 实践) |
| Sinomed | #1 ("颅脑损伤"[不加权:扩展] OR "头部损伤, 穿透性"[不加权:扩展] OR "头部损伤, 闭合性"[不加权:扩展]) OR ("重症颅脑损伤"[常用字段:智能] OR "重症颅脑创伤"[常用字段:智能] OR "重症颅脑外伤"[常用字段:智能] OR "重型颅脑损伤"[常用字段:智能] OR "严重颅脑损伤"[常用字段:智能] OR "创伤性脑损伤"[常用字段:智能] OR "脑外伤"[常用字段:智能] OR "脑损伤"[常用字段:智能] OR "脑出血"[常用字段:智能])) |
|  | #2 ("营养疗法"[不加权:扩展] OR "营养政策"[不加权:扩展] OR "营养支持"[不加权:扩展]) OR ("营养"[常用字段:智能] OR "营养管理"[常用字段:智能] OR "营养治疗"[常用字段:智能] OR "营养支持"[常用字段:智能] OR "营养疗法"[常用字段:智能] OR "营养方案"[常用字段:智能] OR "肠内营养"[常用字段:智能] OR "肠道营养"[常用字段:智能] OR "鼻饲"[常用字段:智能] OR "管饲"[常用字段:智能] OR "管道喂养"[常用字段:智能] OR "鼻肠管喂养"[常用字段:智能] OR "胃管喂养"[常用字段:智能]) |
|  | #3 ("指南"[不加权:扩展] OR "系统综述"[不加权:扩展] OR "循证实践"[不加权:扩展] OR "Meta分析"[不加权:扩展]) OR ("系统综述"[常用字段:智能] OR "指南"[常用字段:智能] OR "证据总结"[常用字段:智能] OR "Meta分析"[常用字段:智能] OR "实践"[常用字段:智能]) |
|  | #1 AND #2 AND #3 |
| VIP Database | (重症颅脑损伤 OR 重症颅脑创伤 OR 重症颅脑外伤 OR 重型颅脑损伤 OR 严重颅脑损伤 OR 创伤性脑损伤 OR 脑外伤 OR 脑损伤 OR 脑出血) AND (营养 OR 营养管理 OR 营养治疗 OR 营养支持 OR 营养疗法 OR 营养方案 OR 肠内营养 OR 肠道营养 OR 鼻饲 OR 管饲 OR 管道喂养 OR 鼻肠管喂养 OR 胃管喂养) AND (指南 OR 系统综述 OR 证据总结 OR Meta分析 OR 实践) |

Figure S1 Flow diagram of the literature selection process

NICE: National Institute for Health and Care Excellence, GIN: guideline repository, Guidelines International Network, ASPEN: American Society for Parenteral and Enteral Nutrition, ESPEN: European Society for Clinical Nutrition and Metabolism, SCCM: Society of Critical Care Medicine, ESICM: European Society of Intensive Care Medicine, ACS: American College of Surgeons, CMA: Chinese Medical Association, CNKI: China National Knowledge Infrastructure.

Figure S2 Delphi survey processes

**Table S2.** Basic characteristics of the included literature (n=17)

| **Included literature** | **Publication year** | **Source** | **Topic** | **Study type** |
| --- | --- | --- | --- | --- |
| Venkatakrishna^1^ | 2024 | UpToDate | Management of acute moderate and severe traumatic brain injury in adults | Clinical decision |
| Babak^2^ | 2024 | UpToDate | Inpatient management of the adult trauma patient | Clinical decision |
| David^3^ | 2025 | UpToDate | Nutrition support in critically ill adult patients | Clinical decision |
| McClave^4^ | 2016 | ASPEN/SCCM | Provision and Assessment of Nutrition Support Therapy in the Adult Critically Ill Patient | Guideline |
| Blaser^5^ | 2017 | Web of Science | Early enteral nutrition in critically ill patients | Guideline |
| Carney^6^ | 2017 | Yimaitong | Management of Severe Traumatic Brain Injury | Guideline |
| Compher^7^ | 2022 | ASPEN | Provision of nutrition support therapy in the adult critically ill patient | Guideline |
| Singer^8^ | 2023 | ESPEN | ICU Nutrition guideline | Guideline |
| Nakamura^9^ | 2024 | Web of Science | Critical Care Nutrition | Guideline |
| Magtoto^10^ | 2022 | JBI | Head Injury: Nutritional Support | Evidence summary |
| Núñez-Patiño^11^ | 2018 | Web of Science | Comparison of intensive versus conventional insulin therapy in traumatic brain injury | Meta-Analysis |
| Yang^12^ | 2021 | Web of Science | Effect of nutritional support on the clinical outcome of patients with traumatic brain injury | Meta-Analysis |
| Qin^13^ | 2023 | Web of Science | Efficacy of Parenteral Nutrition and Enteral Nutrition Supports in Traumatic Brain Injury | Meta-Analysis |
| Awatara^14^ | 2023 | Web of Science | Hyperglycaemia on Admission-Related Mortality in Patients with Severe Traumatic Brain Injury | Meta-Analysis |
| Lu^15^ | 2018 | Wanfang | Glutamine-enhanced enteral nutrition in the treatment of severe traumatic brain injury | Meta-Analysis |
| Du^16^ | 2021 | CNKI | Impact of enteral nutrition support with probiotics added early after severe craniocerebral trauma on the clinical outcomes of patients | Meta-Analysis |
| Jie^17^ | 2024 | CNKI | Comparison of enteral nutrition safety between nasojejunal tubes and nasogastric tubes in patients with severe traumatic brain injury | Meta-Analysis |

**Table S3.** Quality assessment results of the included guidelines (n=6)

| Included literature | Standardized scores of each domain (%) | | | | | | Number of domains with scores ≥60 | Number of domains with scores ≥30 | Recommendation level |
| --- | --- | --- | --- | --- | --- | --- | --- | --- | --- |
|  | Scope and Purpose | Stakeholder Involvement | Rigour of Development | Clarity of Presentation | Applicability | Editorial Independence |  |  |  |
| McClave^4^ | 98.1 | 64.8 | 69.4 | 77.8 | 54.2 | 100 | 5 | 6 | B |
| Blaser^5^ | 74.1 | 66.7 | 63.2 | 79.6 | 47.2 | 69.4 | 5 | 6 | B |
| Carney^6^ | 96.3 | 74.1 | 80.6 | 77.8 | 63.9 | 100 | 6 | 6 | A |
| Compher^7^ | 96.3 | 88.9 | 84.0 | 90.7 | 73.6 | 100 | 6 | 6 | A |
| Singer^8^ | 64.8 | 57.4 | 80.6 | 87.0 | 65.3 | 83.3 | 5 | 6 | B |
| Nakamura^9^ | 98.1 | 90.7 | 89.6 | 75.9 | 69.4 | 100 | 6 | 6 | A |

**Table S4.** Quality assessment results of the included systematic reviews (n=7)

| Included literature | ① | ② | ③ | ④ | ⑤ | ⑥ | ⑦ | ⑧ | ⑨ | ⑩ | ⑪ |
| --- | --- | --- | --- | --- | --- | --- | --- | --- | --- | --- | --- |
| Núñez-Patiño et al.^[11]^ | Yes | Yes | Yes | Yes | Yes | Yes | Yes | Yes | Yes | Yes | Yes |
| Yanget al.^[12]^ | Yes | Yes | Yes | Yes | Yes | Yes | Yes | Yes | Yes | Yes | Yes |
| Qin et al.^[13]^ | Yes | Yes | Yes | Yes | Yes | Unclear | Yes | Yes | No | Yes | Yes |
| Awatara et al^[14]^ | Yes | Yes | Unclear | Yes | No | Yes | Yes | Yes | No | Yes | Yes |
| Lu et al.^[15]^ | Yes | Yes | Yes | Yes | Yes | Yes | Yes | Yes | Yes | Yes | Yes |
| Du^[16]^ | Yes | Yes | Yes | Yes | Yes | Unclear | Yes | Yes | Yes | Yes | Yes |
| Jie et al.^[17]^ | Yes | Yes | Yes | Yes | Yes | Yes | Yes | Yes | Yes | Yes | Yes |

Notes: ① Clear review question; ② Appropriate inclusion criteria; ③ Appropriate search strategy; ④ Adequate search sources; ⑤ Appropriate appraisal criteria; ⑥ Independent critical appraisal; ⑦ Measures to minimize data extraction errors; ⑧ Appropriate synthesis methods; ⑨ Assessment of publication bias; ⑩ Evidence-based recommendations; ⑪ Appropriate suggestions for future research.

**Reference：**

1.	Venkatakrishna Rajajee M. Management of acute moderate and severe traumatic brain injury in adults. 12-08, 2025. Updated 2024-11-20. Accessed 11-20, 2025. https://www.untodate.com/.

2.	Babak Sarani M, FACS, FCCMNiels Martin, MD, FACS, FCCM. Overview of inpatient management of the adult trauma patient. Updated 2024-11-06. Accessed 11-20, 2025. https://www.untodate.com/.

3.	David Seres M. Nutrition support in critically ill adult patients: Initial evaluation and prescription. 11-20, 2025. Updated 2025-07-09. Accessed 11-20, 2025. https://www.untodate.com/.

4.	McClave SA, Taylor BE, Martindale RG, et al. Guidelines for the Provision and Assessment of Nutrition Support Therapy in the Adult Critically Ill Patient. *Journal of Parenteral and Enteral Nutrition*. 2016;40(2):159-211. doi:https://doi.org/10.1177/0148607115621863

5.	Blaser AR, Starkopf J, Alhazzani W, et al. Early enteral nutrition in critically ill patients: ESICM clinical practice guidelines. *INTENSIVE CARE MEDICINE*. MAR 2017;43(3):380-398. doi:10.1007/s00134-016-4665-0

6.	Carney N, Totten AM, O'Reilly C, et al. Guidelines for the Management of Severe Traumatic Brain Injury, Fourth Edition. *NEUROSURGERY*. JAN 2017;80(1):6-15. doi:10.1227/NEU.0000000000001432

7.	Compher C, Bingham AL, McCall M, et al. Guidelines for the provision of nutrition support therapy in the adult critically ill patient: The American Society for Parenteral and Enteral Nutrition. *JPEN J Parenter Enteral Nutr*. Jan 2022;46(1):12-41. doi:10.1002/jpen.2267

8.	Singer P, Blaser AR, Berger MM, et al. ESPEN practical and partially revised guideline: Clinical nutrition in the intensive care unit. *Clin Nutr*. Sep 2023;42(9):1671-1689. doi:10.1016/j.clnu.2023.07.011

9.	Nakamura K, Yamamoto R, Higashibeppu N, et al. The Japanese Critical Care Nutrition Guideline 2024. *JOURNAL OF INTENSIVE CARE*. MAR 21 2025;13(1)18. doi:10.1186/s40560-025-00785-z

10.	Magtoto LS. Head Injury: Nutritional Support. Evidence Summaries. 2022;

11.	Núñez-Patiño RA, Zorrilla-Vaca A, Rivera-Lara L. Comparison of intensive versus conventional insulin therapy in traumatic brain injury: a meta-analysis of randomized controlled trials. *BRAIN INJURY*. 2018;32(6):693-703. doi:10.1080/02699052.2018.1457181

12.	Yang L, Liao DB, Hou XL, Wang Y, Yang CH. Systematic review and meta-analysis of the effect of nutritional support on the clinical outcome of patients with traumatic brain injury. *ANNALS OF PALLIATIVE MEDICINE*. NOV 2021;10(11):11960-11969. doi:10.21037/apm-21-3071

13.	Qin Y, Liu MX, Guo FB, et al. The Efficacy of Parenteral Nutrition and Enteral Nutrition Supports in Traumatic Brain Injury: A Systemic Review and Network Meta-Analysis. *EMERGENCY MEDICINE INTERNATIONAL*. APR 20 2023;20238867614. doi:10.1155/2023/8867614

14.	Awatara PID, Thirza SQ. Hyperglycaemia on Admission-Related Mortality in Patients with Severe Traumatic Brain Injury: A Systematic Review and Meta-Analysis. *ACTA FACULTATIS MEDICAE NAISSENSIS*. 2023;40(1):69-79. doi:10.5937/afmnai40-34751

15.	Lu H, Tan HJ, Feng RY, Chen Q, Yan H. Meta-analysis of glutamine-enhanced enteral nutrition for the treatment of severe traumatic brain injury. Chinese Journal of Trauma. 2018;34(10):898-905. doi:10.3760/cma.j.issn.1001-8050.2018.10.008

16.	Du TJ. A systematic review and meta-analysis of the impact of early probiotic enteral nutrition support on clinical outcomes in patients following severe traumatic brain injury. Master. Tianjin Medical University; 2021. https://link.cnki.net/doi/10.27366/d.cnki.gtyku.2021.000798

17.	Jie LP, Su L, Li Q, Zhang HP, Lin T. Meta-analysis comparing the safety of nasoenteric tubes and nasogastric tubes in enteral nutrition for patients with severe traumatic brain injury. Chinese Journal of Minimally Invasive Neurosurgery. 2024;28(02):94-101. doi:Cnki:Sun:Zwqx.0.2024-02-007

**Table S5.** Suggestions of the First Round of Expert Consultation

| No | Original items | Expert comment |
| --- | --- | --- |
| **Modified** |  |  |
| 1 | Severe TBI was defined as GCS < 9 | Severe TBI is typically classified as a GCS score of 3–8, whereas moderate TBI is classified as 9–12. Therefore, revising the criterion to GCS ≤ 8 would be more consistent with clinical classification. |
| 2 | Nutritional assessment should include comorbidities, gastrointestinal function, and aspiration risk; traditional serum biomarkers are not recommended. | Acute stress responses following TBI may cause fluctuations in traditional serum biomarkers, which are more likely to reflect inflammatory status than nutritional status alone. It is therefore recommended to revise the statement to “traditional serum biomarkers are not recommended as a primary basis for nutritional assessment,” in order to emphasize the principle of comprehensive evaluation. |
| **3** | Preferentially use nasoenteric feeding via the transgastric jejunal route (mainly jejunal feeding) for patients with sTBI. | It was suggested to use the term postpyloric feeding, as it is more standardized and clinically appropriate. To enhance procedural accuracy and feasibility, the method for confirming tube placement should be specified, and a transitional strategy should be added for clinical practice: when placement of a nasoenteric tube is difficult, feeding may be initiated via a nasogastric tube and converted to a nasoenteric tube as soon as conditions permit. |
| 4 | In all intubated patients receiving enteral nutrition (EN), the head of the bed should be elevated to 30°–45°. | It was suggested to specify that this measure does not apply to patients with contraindications to head-of-bed elevation, in order to improve clinical safety and applicability |
| 5 | Blood glucose should be measured initially (after admission or after the initiation of artificial nutrition) and at least every 4 hours during the first 48 hours; more frequent monitoring is required in unstable patients. | It was suggested to further specify the monitoring frequency after blood glucose becomes stable, with a general recommendation of every 6 hours, in order to make the monitoring protocol more standardized and clinically practical. |
| 6 | Early initiation of supplemental parenteral nutrition is not recommended in patients with insufficient enteral nutrition. | It was suggested that the expression “early initiation” was not sufficiently specific. According to current guideline recommendations, the timing of supplemental parenteral nutrition should be expressed as 7–10 days, rather than using a vague or overly restrictive statement, to improve clarity and consistency with the evidence. |
| 7 | Nutritional tolerance should be monitored dynamically; when necessary, lactate levels, gastric residual volume (GRV), and imaging examinations should be used for comprehensive assessment. | It was suggested to clarify how nutritional tolerance should be monitored, including the monitoring frequency and the basis for assessment or scoring, in order to make the item more standardized and clinically applicable. |
| 8 | Glutamine should be supplemented at 0.2–0.3 g/kg/d during the first 5 days after injury in patients with sTBI. | Given the ongoing controversy regarding the benefits of glutamine in critically ill patients, particularly those with multiple organ dysfunction, it was recommended that this item be revised to specify that glutamine should be used selectively and only in patients with preserved liver and renal function. |
| **Merged** |  |  |
| 1 | (1) Once nutritional support is initiated, patient tolerance and complications must be monitored. | It was suggested to merge these two items, as both focus on the implementation and monitoring of nutritional support. |
|  | (2) A nurse-led enteral nutrition protocol is recommended to increase enteral nutrition delivery and reduce the incidence of hospital-acquired infections. |  |
| 2 | (1) Immunomodulating nutritional formulas should be used on the basis of standard enteral nutrition in patients with sTBI. The formula may include arginine, glutamine, EPA, DHA, nucleotides, lysine, and other components. | To enhance the structural coherence of the protocol, "probiotics" and "immunomodulating formulas" were consolidated into a single item. This integration avoids a fragmented presentation of nutritional interventions and better reflects the synergistic role of adjunctive components in enteral nutrition. |
|  | (2) Probiotics are recommended as a component of enteral nutrition in patients with sTBI. |  |
| **Deleted** |  |  |
| ~~1~~ | Patients hospitalized for more than 48 hours are considered at risk of malnutrition. | This item relies solely on duration of hospitalization to define nutritional risk, which is not sufficiently rigorous. Nutritional risk should be identified through comprehensive assessment; therefore, deletion of this item is recommended.  (Note: Mean score did not meet the predefined criterion; the core concept was integrated into the revised item on energy requirement assessment. ) |
| Split |  |  |
| 1 | Blood glucose should be monitored every 4 hours during the first 48 hours after admission or initiation of artificial nutrition. For patients with instability or marked glucose fluctuations, the monitoring frequency should be increased as appropriate. Once blood glucose becomes stable, monitoring may be reduced to every 6 hours. If insulin therapy is initiated, blood glucose should be monitored every 0.5–1 hour during the initial adjustment period, and may be reduced to every 2–4 hours after the target range is achieved and the insulin dose becomes stable. | It was suggested to separate the blood glucose monitoring frequency during insulin initiation from the general monitoring schedule, so as to present the item more clearly and improve its readability. |
| 2 | In patients with shock, low-dose enteral nutrition may be initiated within 24–48 hours after adequate fluid resuscitation and achievement of relative hemodynamic stability (e.g., norepinephrine dose ≤ 0.1–0.2 μg/kg/min with a stable or decreasing trend), with an initial rate of 10–20 mL/h; in patients with sTBI receiving therapeutic hypothermia, low-dose enteral nutrition may be started initially and then gradually advanced according to the rewarming process and feeding tolerance. | It was suggested to split this item into two separate items, as nutrition initiation in patients with shock and in those undergoing therapeutic hypothermia represent two different clinical conditions with distinct initiation criteria and management considerations. Splitting the item would improve structural clarity. |
| 3 | Standard enteral nutrition can be supplemented with immunomodulating formulas based on clinical assessment. These formulas may incorporate arginine, glutamine, nucleotides, and omega-3 fatty acids, with probiotics added for selected patients. For those with stable hepatic and renal function, glutamine supplementation (0.2–0.3 g/kg/d) is recommended within the first 5 days post-injury, with dosages adjusted according to individual tolerance. | It was suggested that the glutamine-related recommendation be separated into an independent item, so as to avoid combining formula selection and dosage regimen within a single item and to allow the focus of the content to be more concentrated. |

**Table S6.** Detailed Nutritional Support Management Protocol for Patients with Severe Traumatic Brain Injury

| Recommendation (Implementation plan) | Feasibility Score (Mean± SD) | CV | Importance Score (Mean± SD) | CV |
| --- | --- | --- | --- | --- |
| **Ⅰ. Organizational Support and Team Management** | 4.73±0.46 | 0.10 | 4.93±0.26 | 0.05 |
| 1. Multidisciplinary Team Development: A multidisciplinary nutritional support team should be established, consisting of intensivists, neurosurgeons, clinical specialist nurses, dietitians, and rehabilitation therapists. | 4.53±0.52 | 0.11 | 4.93±0.26 | 0.05 |
| 2. Standardized Procedures, Training, and Quality Control: Standardized procedures for nutritional support should be established, with clear delineation of responsibilities for each role; nutritional support training and quality control should be conducted for the team every two weeks. | 4.93±0.26 | 0.05 | 5.00±0.00 | 0.00 |
| **Ⅱ. Nutritional Screening, Pre-initiation Assessment, and Decision-making** | 4.67±0.49 | 0.10 | 5.00±0.00 | 0.00 |
| A. **Nutritional Risk Screening** | 4.93±0.26 | 0.05 | 5.00±0.00 | 0.00 |
| 3. Nutritional risk screening should be performed within 24 h of admission for new patients with sTBI (GCS ≤ 8). Rescreening is required after one week of hospitalization or upon acute clinical changes (e.g., secondary surgery, severe infection). | 4.73±0.46 | 0.10 | 4.80±0.41 | 0.09 |
| 4. Screening should be conducted using the NRS-2002 or NUTRIC score. | 5.00±0.00 | 0.00 | 5.00±0.00 | 0.00 |
| B. **Multidimensional Nutritional Assessment and Contraindication Screening** | 5.00±0.00 | 0.00 | 5.00±0.00 | 0.00 |
| 5. Monitor blood glucose levels in all sTBI patients upon admission, and dynamically evaluate glycemic metabolism in conjunction with the implementation of artificial nutrition. | 5.00±0.00 | 0.00 | 4.93±0.26 | 0.05 |
| 6. Conduct a comprehensive nutritional assessment based on contraindications, comorbidities, gastrointestinal function, aspiration risk, and metabolic status; the use of traditional serum biomarkers as the sole indicator for nutritional assessment is not recommended. | 4.60±0.51 | 0.11 | 4.93±0.26 | 0.05 |
| 7. Perform a systematic evaluation prior to initiating nutritional support to exclude contraindications for enteral nutrition (e.g., intestinal obstruction, hemodynamic instability) and parenteral nutrition (e.g., uncontrolled hyperglycemia, severe electrolyte imbalances). | 4.87±0.35 | 0.07 | 5.00±0.00 | 0.00 |
| C. **Timing of Nutrition Initiation** | 4.67±0.49 | 0.10 | 5.00±0.00 | 0.00 |
| 8. For sTBI patients without contraindications to EN, early initiation of EN is recommended within the first 48 hours of admission. | 4.53±0.52 | 0.11 | 4.93±0.26 | 0.05 |
| 9. In patients with shock, low-dose EN (initial rate of 10-20 mL/h) should be initiated within 24–48 hours after achieving hemodynamic stability following fluid resuscitation (e.g., norepinephrine dose ≤0.1-0.2μg/kg/min with a stable or decreasing trend). | 4.33±0.49 | 0.11 | 4.87±0.35 | 0.07 |
| 10. For sTBI patients undergoing therapeutic hypothermia, EN should be initiated at a low dose, with gradual escalation based on patient tolerance and the rewarming process. | 4.67±0.49 | 0.10 | 4.67±0.49 | 0.10 |
| 11. For patients with contraindications to EN or those unable to meet nutritional goals via the enteral route, the timing of PN initiation should be evaluated based on nutritional risk, disease severity, and clinical tolerance. | 4.07±0.59 | 0.15 | 4.13±0.35 | 0.09 |
| 12. For patients requiring early parenteral support, PN may be initiated within 36–48 hours of admission. | 4.07±0.59 | 0.15 | 4.20±0.41 | 0.10 |
| **Ⅲ. Nutritional Interventions** | 4.40±0.63 | 0.14 | 4.87±0.35 | 0.07 |
| D. **Feeding Route and Delivery Method** | 4.87±0.35 | 0.07 | 5.00±0.00 | 0.00 |
| 13. Post-pyloric feeding is preferred. In patients with contraindications to nasal tube insertion (e.g., skull base fractures, nasal trauma), the oral route may be considered first. Tube position should be confirmed by X-ray or bedside ultrasound after placement. | 4.20±0.68 | 0.16 | 4.87±0.35 | 0.07 |
| 14. Continuous EN should be delivered using a controllable feeding pump. | 5.00±0.00 | 0.00 | 4.93±0.26 | 0.05 |
| E. **Energy Requirements and Target Setting** | 4.53±0.52 | 0.11 | 4.87±0.35 | 0.07 |
| 15. IC is preferred for measuring EE. If IC is unavailable, EE may be estimated using VCO_2_ obtained from the ventilator according to REE = VCO_2_× 8.19, or VO_2_ obtained from a pulmonary artery catheter. If IC, VCO_2_, and VO_2_ are unavailable, a weight-based estimate of 25–30 kcal/kg/d based on actual or adjusted body weight, or validated predictive equations may be used initially, with adjustment according to the individual patient’s age, body size, disease severity and metabolic status. | 4.47±0.74 | 0.17 | 4.80±0.41 | 0.09 |
| 16. Nutritional delivery should follow a step-by-step progression, with baseline caloric requirements achieved within 5 to 7 days post-injury. | 4.00±0.65 | 0.16 | 4.80±0.41 | 0.09 |
| 17. For sTBI patients at high nutritional risk (e.g., NRS 2002 ≥ 5 or NUTRIC score ≥ 5) or with severe malnutrition, aim to achieve >80% of target energy and protein requirements within 48–72 hours. | 4.00±0.53 | 0.13 | 4.00±0.38 | 0.09 |
| 18. The target protein intake is 1.2–2.0 g/kg/d, with dynamic adjustments based on renal function, metabolic status, and clinical tolerance. | 4.27±0.46 | 0.11 | 4.80±0.41 | 0.09 |
| F. **Supplemental PN Strategy** | 4.47±0.52 | 0.12 | 4.67±0.49 | 0.10 |
| 19. Initiation of SPN should be based on a comprehensive assessment of nutritional risk, disease severity, and EN tolerance. Early initiation of SPN is not recommended for patients already receiving partial EN. | 4.20±0.41 | 0.10 | 4.40±0.51 | 0.12 |
| 20. For sTBI patients failing to meet >60% of energy and protein requirements via the enteral route, SPN may be considered within 7–10 days of admission, with the amount and composition individualized according to nutritional risk, disease severity, remaining energy and protein deficits, metabolic status, organ function, and clinical tolerance. | 4.40±0.51 | 0.12 | 4.33±0.49 | 0.11 |
| G. **Selection of Immunomodulatory and Enteral Formulas** | 4.40±0.51 | 0.12 | 4.47±0.52 | 0.12 |
| 21. Immunomodulating formulas may be combined with standard EN based on the patient’s clinical status and assessment. | 4.47±0.52 | 0.12 | 4.47±0.52 | 0.12 |
| 22. Immunomodulating components may include arginine, glutamine, omega-3 fatty acids, and nucleotides; probiotics may be added for selected patients as appropriate. | 4.00±0.38 | 0.09 | 4.07±0.26 | 0.06 |
| 23. For sTBI patients with preserved hepatic and renal function, glutamine may be supplemented during the first 5 days post-injury at a recommended dose of 0.2-0.3g/kg/d, with adjustments based on clinical status and tolerance. | 4.07±0.59 | 0.15 | 4.00±0.53 | 0.13 |
| 24. Routine fiber supplementation is not recommended for sTBI patients with severe gastrointestinal dysmotility or evident feeding intolerance. | 4.80±0.41 | 0.09 | 4.60±0.51 | 0.11 |
| **Ⅳ.Implementation, Monitoring, and Complication Management** | 4.80±0.41 | 0.09 | 5.00±0.00 | 0.00 |
| H. **Standardized Implementation and Position Management** | 5.00±0.00 | 0.00 | 5.00±0.00 | 0.00 |
| 25. Implement a nurse-led enteral nutrition protocol; feeding tolerance and related complications should be continuously monitored following the initiation of nutritional support. | 4.93±0.26 | 0.05 | 5.00±0.00 | 0.00 |
| 26. In the absence of contraindications (e.g., uncorrected hemorrhagic shock, unstable spinal injury), the head of the bed should be elevated to 30°–45° for all intubated patients receiving EN to reduce the risk of reflux and aspiration. | 5.00±0.00 | 0.00 | 5.00±0.00 | 0.00 |
| I. **Assessment of Feeding Tolerance and Gastric Residual Volume Monitoring** | 4.80±0.41 | 0.09 | 5.00±0.00 | 0.00 |
| 27. Dynamic tolerance monitoring: Evaluate abdominal signs and gastrointestinal symptoms (e.g., nausea, vomiting, diarrhea) every 4 hours using a standardized assessment scale. When necessary, integrate lactate levels, GRV, and imaging findings for a comprehensive determination. | 4.60±0.51 | 0.11 | 4.87±0.35 | 0.07 |
| 28. GRV monitoring: Monitor GRV using a 50/60 mL syringe via the gastric/enteral tube only when symptoms such as abdominal distension, nausea, or vomiting occur. Routine GRV monitoring is not recommended for patients without evident gastrointestinal distress. | 4.67±0.49 | 0.10 | 4.60±0.51 | 0.11 |
| J. **Blood Glucose Monitoring and Control** | 4.67±0.49 | 0.10 | 5.00±0.00 | 0.00 |
| 29. Monitor blood glucose every 4 hours upon admission or within the first 48 hours of artificial nutrition. For patients with diabetes, steroid therapy, osmotherapy, significant glucose fluctuations, or unstable neurological/metabolic status, monitoring frequency should be increased; once stable, frequency may be reduced to every 6 hours. | 4.73±0.46 | 0.10 | 5.00±0.00 | 0.00 |
| 30. During the initial titration phase of insulin therapy, monitor blood glucose every 0.5-1 hour. Once the target range is achieved and the insulin dose is stabilized, monitoring frequency can be adjusted to every 2-4 hours. | 4.87±0.35 | 0.07 | 4.93±0.26 | 0.05 |
| 31. Intensive glucose control is not recommended for sTBI patients. Blood glucose should be maintained within the range of 140-180 mg/dL (7.8-10.0 mmol/L); insulin intervention should be initiated if levels exceed the target range. | 4.33±0.72 | 0.17 | 5.00±0.00 | 0.00 |
| K. **Prevention of Refeeding Syndrome and Management of Feeding Intolerance** | 4.47±0.52 | 0.12 | 4.60±0.51 | 0.11 |
| 32. Measure serum electrolytes daily during the first week of enteral nutrition. If hypophosphatemia occurs, monitor 2–3 times daily; restrict energy intake for 48 hours before gradually resuming full delivery. | 4.07±0.70 | 0.17 | 4.67±0.62 | 0.13 |
| 33. If GRV >500 mL within 6 hours or significant intolerance occurs, enteral nutrition should be suspended or slowed. Evaluate the underlying cause and consider prokinetic agents (e.g., erythromycin, metoclopramide) based on the patient's condition. | 4.67±0.49 | 0.10 | 4.93±0.26 | 0.05 |

Notes: NRS-2002: Nutritional Risk Screening 2002; NUTRIC: Nutrition Risk in the Critically Ill; EN: Enteral Nutrition; PN: parenteral nutrition; IC: Indirect calorimetry; EE: energy expenditure; REE: resting energy expenditure; SPN: supplemental parenteral nutrition; GRV: Gastric residual volume.
